# Role of ureides in source-to-sink transport of photoassimilates in non-fixing soybean

**DOI:** 10.1093/jxb/eraa146

**Published:** 2020-03-19

**Authors:** Sandi Win Thu, Ming-Zhu Lu, Amanda M Carter, Ray Collier, Anthony Gandin, Ciera Chenoa Sitton, Mechthild Tegeder

**Affiliations:** 1 School of Biological Sciences, Washington State University, Pullman, WA, USA; 2 Michigan State University, USA

**Keywords:** Amino acid assimilation, legume, nitrogen and carbon metabolism, phloem loading, photoassimilate partitioning, seed development, source-to-sink transport, soybean, ureide transporter function

## Abstract

Nitrogen (N)-fixing soybean plants use the ureides allantoin and allantoic acid as major long-distance transport forms of N, but in non-fixing, non-nodulated plants amino acids mainly serve in source-to-sink N allocation. However, some ureides are still synthesized in roots of non-fixing soybean, and our study addresses the role of ureide transport processes in those plants. In previous work, legume ureide permeases (UPSs) were identified that are involved in cellular import of allantoin and allantoic acid. Here, *UPS1* from common bean was expressed in the soybean phloem, which resulted in enhanced source-to-sink transport of ureides in the transgenic plants. This was accompanied by increased ureide synthesis and elevated allantoin and allantoic acid root-to-sink transport. Interestingly, amino acid assimilation, xylem transport, and phloem partitioning to sinks were also strongly up-regulated. In addition, photosynthesis and sucrose phloem transport were improved in the transgenic plants. These combined changes in source physiology and assimilate partitioning resulted in increased vegetative growth and improved seed numbers. Overall, the results support that ureide transport processes in non-fixing plants affect source N and carbon acquisition and assimilation as well as source-to-sink translocation of N and carbon assimilates with consequences for plant growth and seed development.

## Teaser

UPS1 phloem loading function represents a bottleneck in long-distance ureide transport, affects N and C acquisition and metabolism, and leads to rebalancing of whole-plant amino acid and sucrose partitioning.

## Introduction

Soybean (*Glycine max* L. Merr.) plants can fix atmospheric nitrogen (N) in a symbiotic relationship with bacteria that reside in root nodules. The main products of this biological N fixation process are the ureides allantoin and allantoic acid, which serve as long-distance N transport forms allocated in the xylem to the shoot (Herridge, 1982; Kouchi and Higuchi, 1988; Atkins and Smith, 2007). In soils with low organic matter and residual mineral N, fixation provides the majority of N acquired by legumes. However, many soils contain high amounts of organic matter and rather large pools of mineral N. In these soils, the inorganic N decreases nodule formation and N fixation so that N fixation may provide relatively little N for soybean growth and reproduction (Gibson and Harper, 1985; Imsande 1986; Yamashita *et al.*, 2019). It has been predicted that, dependent on the amount of soil N, microbial activity, and available water, 25–99% of the seasonal N may derive from N uptake from the soil compared with N fixation (Weber, 1966; Harper, 1974, 1987). Therefore, understanding the regulatory mechanisms of root N acquisition, assimilation, and partitioning, and their inter-relationship with carbon (C) fixation and metabolism in non-fixing soybean plants is essential for the development of strategies aimed to enhance soybean productivity.

Non-nodulated or non-fixing soybean plants take up nitrate and ammonium from the soil that is reduced in roots to amino acids, which then serve as main long-distance transport forms of N allocated in the xylem to the shoot (Oaks, 1992; Ueda *et al.*, 2008). Non-fixing soybean plants also synthesize allantoin and allantoic acid via the *de novo* purine synthesis and degradation pathway (Todd *et al.*, 2006; Werner and Witte, 2011), but the ureide levels are relatively low compared with amino acids and may only contribute 1–10% to the N that is allocated in the xylem to photosynthetically active source leaves (Matsumoto *et al.*, 1977; McClure and Israel, 1979; McNeil and LaRue, 1984; Oaks, 1992; Do Amarante *et al.*, 2006). Following their synthesis in roots, ureides (and amino acids) are mostly allocated in the xylem to transpiring source leaves (Atkins and Beevers, 1990; Atkins and Smith, 2000). However, along the translocation pathway, some ureides may be transferred from the xylem to the phloem for immediate supply of sinks (Pate *et al.*, 1975; Sharkey and Pate, 1975; van Bel, 1984, 1990), or they are transiently stored in the stem and petioles (Herridge *et al.*, 1978). Additionally, ureides can travel in the xylem sap directly to sink tissues (Layzell and LaRue, 1982). The allantoin and allantoic acid in source leaves either derive from roots via the xylem or are produced through breakdown of purine nucleotides (Todd *et al.*, 2006; Werner and Witte, 2011). Ureides can be temporarily stored, which occurs in soybean leaves particularly in bundle sheath cells and the paraveinal mesophyll (Franceschi and Giaquinta, 1983; Franceschi *et al.*, 1983; Costigan *et al.*, 1987). Alternatively, they are catabolized to release ammonia for re-assimilation into amino acids (Winkler *et al.*, 1987; Todd *et al.*, 2006; Werner and Witte, 2011), some of which may be stored by incorporation into vegetative storage proteins (VSPs; Franceschi and Giaquinta, 1983; Staswick *et al.*, 1991). Ureides are further loaded into the leaf phloem to supply developing sinks, including leaves, root tips, pods, or seeds, with N (Atkins and Beevers, 1990). Ultimately, the N is released from ureides in sink tissues and used for synthesis of amino acids essential for physiological functions, and growth and development of sink organs (Coker and Schaefer, 1985; Todd *et al.*, 2006).

Movement of ureides from legume roots or nodules to leaves and finally to sinks requires the function of plasma membrane-localized proteins that regulate the partitioning of the organic N. Recent studies have identified legume UPS1 ureide permeases that mediate cellular import of allantoin and allantoic acid (Pélissier *et al.*, 2004; Pélissier and Tegeder, 2007; Collier and Tegeder, 2012). Overexpression of the common bean (*Phaseolus vulgaris* L.) *PvUPS1* transporter gene in the nodule cortex and vascular endodermis cells of N-fixing soybean plants resulted in increased movement of allantoin and allantoic acid from nodules to shoot, with positive effects on plant growth and seed development (Carter and Tegeder, 2016). In the non-legume species rice (*Oryza sativa* L.), an activation-tagging line overexpressing *OsUPS1* throughout the plant showed improved growth under N limitation (Redillas *et al.*, 2019). The authors also used constitutive overexpression of *OsUPS1*, leading to increased allantoin levels in leaves, stems, and roots, as well as an *OsUPS1* RNAi line that accumulated allantoin in roots. Since *UPS1* is expressed throughout the plant and since it was overexpressed or repressed in the whole plant body, it is difficult to conclude what caused the reported changes in growth and ureide levels. However, as suggested, OsUPS1 may affect the N status of the rice plants through both enhanced synthesis and partitioning of allantoin (Redillas *et al.*, 2019).

The current study addresses the role of ureide partitioning processes in the performance of non-nodulated, non-fixing soybean using plants that express the *PvUPS1* transporter gene in the phloem throughout the plant. We analyzed whether or how ureide source-to-sink transport contributes to sink N nutrition and development. Further, recent studies support that ureides may act as signals controlling plant responses to environmental stresses, including the regulation and coordination of primary metabolism and plant growth (Winkler *et al.*, 1988; Nikiforova *et al.*, 2005; Nakagawa *et al.*, 2007; Brychkova *et al.*, 2008; Werner *et al.*, 2013; Coneva *et al.*, 2014; Redillas *et al.*, 2019). While the mechanism behind this is still unclear, it has been suggested that ureide compartmentation and transport processes are involved. Therefore, effects of ureide partitioning processes on N and C metabolism and phloem source-to-sink transport as well as on sink development were examined in the transgenic *UPS1* soybean plants. Overall, our data hint at a complex inter-relationship between ureide transport and N/C metabolism, N/C assimilate source-to-sink transport, and seed productivity, and lend further support for a regulatory role of ureides in basic plant physiological processes.

## Materials and methods

### Plant material and growth conditions

Non-nodulated soybean (cultivar Hutcheson; Buss *et al.*, 1988) wild-type as well as transgenic plants expressing either the *PvUPS1* ureide transporter gene from *P. vulgaris* (Pélissier *et al.*, 2004) under control of the corresponding *PvUPS1* promoter (Carter and Tegeder, 2016) or a *PvUPS1* promoter–β-glucuronidase (GUS) gene construct (Jefferson *et al.*, 1987; Collier and Tegeder, 2012) were analyzed. Individual plants were grown in a growth chamber in 3.75 liter pots and in the greenhouse in 26.5 liter pots containing potting mix (SunGro Horticulture Inc., Bellevue, WA, USA), comprised of peat (60%), pumice (20%), and sand (20%). They were exposed to a 16 h photoperiod at a light intensity of 1000 µmol photons m^–2^ s^–1^ photosynthetically active radiation (PAR). The day/night temperature and relative humidity were 26 °C/21 °C and 50%/70%, respectively. Growth chamber plants were watered twice daily, with a total of ~1 liter of water, and fertilized once a week with 250 ml per pot of 2.5 g l^–1^ 20:20:20 (N:P:K) fertilizer (J.R. Peters, Allentown, PA, USA). Roots, stems, fully expanded, photosynthetically active source leaves, and developing sink leaves, as well as xylem sap and phloem exudates, were collected 36 days after planting (DAP). Root samples consisted of a mix of tap and lateral roots collected from the middle part of the root system to the tip. Stem tissues were taken between the fourth and eighth inserted leaves counting from the bottom of the plant. The source leaf and phloem exudate samples derived from the fifth and sixth leaf, and sink leaves from the ninth and tenth leaf. The liquid and tissue samples were immediately frozen in liquid N and stored at –80 °C or the tissues were ground and lyophilized. Greenhouse plants were watered daily to saturation, fertilized once a week with 2.5 g l^–1^ Peters 20-20-20, and grown until desiccation. Dried pods and seeds were collected around 275 DAP.

### Construct preparation for plant transformation and localization studies

The *PvUPS1* promoter–*GUS* construct previously described (Collier and Tegeder, 2012) was stably expressed in soybean. Transgenic *PvUPS1* promoter–*GUS* lines were kindly produced at the Plant Tissue Culture and Transformation Laboratory at the Donald Danforth Plant Sciences Center in Saint Louis, MO, USA and screened for homozygosity using standard procedures.

For membrane localization studies, *PvUPS1* transporter cDNA–green fluorescent protein (GFP) gene fusion constructs were prepared using an MBCS plasmid vector (Collier *et al.*, 2005) containing the *Super Ubiquitin* (*SU*; Perera and Rice, 2002) promoter, an *SU* intron, and a full-length *GFP5* gene (Siemering *et al.*, 1996). The *Pac*I restriction sites were substituted by *Sda*I sites and the *GFP* gene was replaced with *PvUPS1* (*Bam*HI/*Sac*I). An adaptor was produced with a pair of reverse complementary primers (TTCGGATCCACTTCTGCTGCTGGTTCTGCTGCTGGTTCTGCTATGTACGTACT and AAGTACGTACATAGCAGAACCAGCAGCAGAACCAGCAGCAGAAGTGGATCCGA) and inserted in the *Bam*HI/*Eco*105I site upstream of the *PvUPS1* cDNA. A *GFP5* gene without a stop codon was cloned into the *Bam*HI site upstream of the adaptor, resulting in the final cassette containing *SU* promoter–*SU* intron–*GFP5*–adaptor–*PvUPS1* cDNA–*NOS* terminator. The *GFP*–*PvUPS1* fusion cassette was transferred as an *Asc*I fragment into an MBCS binary vector (Collier *et al.*, 2005).

### Histochemical analysis of *PvUPS1* promoter*–GUS* lines

Soybean leaf, stem, and root tissues were analyzed by the GUS staining procedure according to Jefferson *et al.* (1987) to determine the location of *PvUPS1* expression. The samples were incubated overnight at 37 °C and subsequently cleared of chlorophyll with 95% (v/v) ethanol. Tissue or tissue hand sections were analyzed with a stereoscopic light microscope (Wild, HeerBrugg, Switzerland) and a Leica DM LFSA light microscope (Leica, Wetzlar, Germany), respectively.

### Subcellular localization of *PvUPS1*

For subcellular localization of *GFP*–*PvUPS1*, the *Nicotiana benthamiana* Domin leaf infiltration method was used (Sparkes *et al.*, 2006). *Agrobacterium rhizogenes* 18r12v carrying the *GFP–PvUPS1* transporter fusion constructs was co-infiltrated with *A. tumefaciens* strain GV3101 pMP90 harboring the p19 protein gene of *Tomato bushy stunt virus* to repress silencing in plant cells (Voinnet *et al.*, 2003) as well as with *A*. *rhizogenes* 18r12v containing aquaporin *AtPIP2A* fused to *mCherry* (Arabidopsis Biological Resource Center, stock number CD3-1007; Nelson *et al.*, 2007) that localizes to the plasma membrane (Cutler *et al.*, 2000; Shaner *et al.*, 2004). Leaf tissue was analyzed by confocal microscopy (Leica, Wetzlar, Germany). Sodium chloride (1 M) was added to some specimens to induce plasmolysis and to more clearly visualize plasma membrane localization.

### RNA expression analysis

Total RNA was isolated from roots, stems, and source leaves (Santiago and Tegeder, 2016) using up to six biological replicates. RNA extraction was performed with TRIzol reagent (Thermo Fischer Scientific, Waltham, MA, USA) following the manufacturer’s protocol and as described by Chomczynski and Sacchi (1987). Samples were treated with TURBO DNase (Thermo Fisher Scientific), and first-strand cDNA synthesis was performed using MMLV reverse transcriptase (Thermo Fisher Scientific). *PvUPS1* expression was analyzed by a semi-quantitative reverse transcription–PCR (RT–PCR) approach followed by gel electrophoresis; Tan *et al.*, 2008). To determine expression of ureide transport and N metabolism genes, quantitative real-time RT–PCR (qRT–PCR) was performed following Zhang *et al.* (2010) and by using a 1:10 cDNA dilution. Primers were designed along non-conserved DNA regions to ensure gene-specific amplification. For gene accessions, primer information, and references, see Supplementary Table S1 at *JXB* online. Experiments were performed with at least four biological replicates (see figure legends) using an Applied Biosystems 7500 Fast Thermal cycler (Foster City, CA, USA) to produce threshold (C_T_) values (Sanders *et al.*, 2009). *SKIP16* (*SKP1/Ask-Interacting Protein 16*) and *ACT11* (*ACTIN11*) were used as reference for normalizing gene expression data (Hu *et al.*, 2009; Ma *et al.*, 2013). Fold changes in gene expression were determined by comparing the C_T_ values with the reference genes using the 2^−ΔΔCT^ method (Livak and Schmittgen, 2001).

### Xylem sap and phloem leaf exudate collection

Xylem sap was collected using the positive root pressure technique as described in Pélissier and Tegeder (2007). Plants were watered at the beginning of the light period and then again 5 min prior to stem decapitation at 1 cm above the soil surface. Xylem sap oozing during the first 5 min was discarded and the subsequent sap was collected for 20 min and stored at –80 °C. Undiluted xylem sap samples were used for analyses of total ureides, allantoin, allantoic acid, and free amino acids.

Leaf phloem exudates were collected from two leaflets (fifth and sixth when counting from the bottom). Leaves were cut at the petiole base and transferred to a tube containing 2 ml of 20 mM EDTA at pH 7.3 (King and Zeevaart, 1974; Urquhart and Joy, 1981). Leaves were wrapped with wet paper towels and kept in the dark in a humid chamber for 4 h. Undiluted exudates were directly used for ureide analysis. Alternatively, the EDTA in the exudates was precipitated with 1/10 volume of 1 M HCl at –20 °C for 12 h. After centrifugation at 18 000 *g* for 20 min at 4 °C, the supernatant was used for amino acid and sucrose analyses.

### Analysis of ureides and amino acids

Total ureides, allantoin, and allantoic acid levels were analyzed according to Collier and Tegeder (2012) using 5 mg of lyophilized tissues or 25 µl and 300 µl of undiluted xylem sap and leaf phloem exudates, respectively. Amino acids were extracted and derivatized with 4-fluoro-7-nitro-2,1,3-benzoxadiazole (NBD-F) as previously described (Aoyama *et al.*, 2004), and HPLC was performed following Lu *et al.* (2020).

### Analysis of soluble protein, sucrose, and elemental N

Lyophilized tissues from stems (2 mg), source leaves (3 mg), and dry seeds (1 mg) from at least five plants (*n* ≥5) were used for analysis of soluble proteins. Proteins were extracted following Zhang *et al.* (2015). Diluted stem (1:10), source leaf (1:100), and seed (1:100) extracts were used to determine the protein amounts with the NanoOrange protein quantification kit according to the manufacturer’s protocol (Invitrogen, Carlsbad, CA, USA) and a Bio-Tek Synergy HT microplate reader (excitation, 480 nm; emission, 590 nm; Winooski, VA, USA). Sucrose levels in leaf phloem exudates were determined as described (Zhang *et al.*, 2015). Total elemental N content was analyzed according to Sanders *et al.* (2009).

### Photosynthesis measurements

Net photosynthetic rates were determined using 32-day-old plants and the LI-6400XT system (LI-COR Biosciences, Lincoln, NE, USA). Photosynthetic rates were measured on fully expanded leaves in positions 4 and 5 at 300 μmol quanta m^−2^ s^−1^ PAR light intensity. The oxygen and CO_2_ partial pressure were maintained at 18.4 kPa and 37.2 Pa, respectively. The leaf temperature was kept at 25 °C, and the relative humidity was adjusted to maintain a vapor pressure deficit between 0.9 kPa and 1.3 kPa. Prior to all measurements, leaves were acclimated for 20 min or until steady state was reached.

### Collection of yield data

Dry pods were collected from individual plants and sorted by number of seeds per pod. The pods generally contained one, two, or three seeds. Seed weight was determined from 9–12 plants using three pools of 50 seeds per plant.

### Statistical analysis

Molecular, biochemical, physiological, and growth data were collected from up to 12 individual *UPS1-OE1*, *UPS1-OE2*, and wild-type plants. The biological repeats for each analysis can be found in the figure legends. Data are presented as mean ±SD of biological repetitions and were analyzed by one-way ANOVA or Student’s *t*-tests using SigmaPlot 11.0 (Systat Software, Chicago, IL, USA). Results are presented in bar graphs, and the percentage change in transgenic versus wild-type plants is indicated by numbers above the columns. Small, moderate, and large statistically significant changes are specified (*P≤0.05, **P≤0.01, ***P≤0.001).

## Results

### The *PvUPS1* promoter targets gene expression to the phloem in soybean plants

**Fig. 1.**
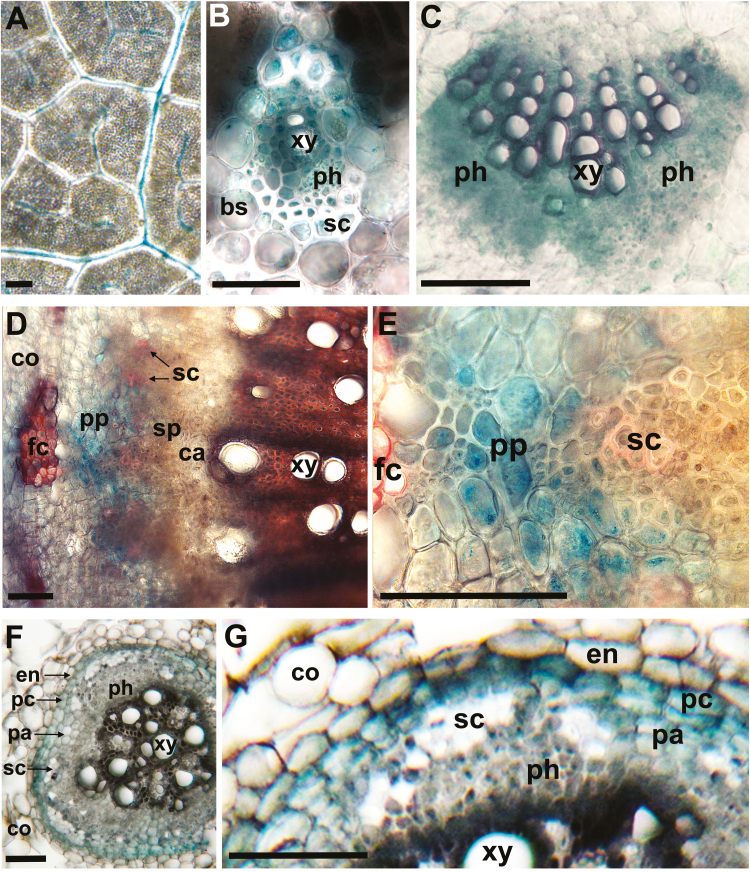
*PvUPS1* promoter–*GUS* analysis in soybean. (A–C) Source leaves. (A) GUS staining is evident in the leaf vasculature of (B) minor and (C) major veins, including the phloem, bundle sheath cells, and vascular parenchyma. Scale bars=1 mm in (A), 50 μm in (B) and (C). (D and E) Stem cross-sections. Staining is seen in the phloem and surrounding cells. Scale bars=250 μm. (F and G) Root cross-sections. *GUS* expression is reported in the endodermis, pericycle, phloem, and vascular parenchyma. Scale bars=50 μm. Bundle sheath cells, bs; cambium, ca; cortex, co; endodermis, en; pericycle, pc; phloem fiber cap, fc; parenchyma, pa; phloem, ph; primary phloem, pp; secondary phloem, sp; sclerenchyma cells, sc; xylem, xy.

Stable transgenic soybean plants expressing the common bean ureide transporter gene *PvUPS1* under control of the *PvUPS1* promoter have previously been produced to determine UPS1 function in nodules (Carter and Tegeder, 2016). Further, using transient expression in composite soybean plants that develop transgenic nodulated roots and a non-transgenic shoot, it has been shown that the *PvUPS1* promoter targets gene expression to the inner cortex and vascular endodermis cells of soybean nodules (Collier and Tegeder, 2012). However, the *PvUPS1* promoter activity in other plant tissues was not resolved. We now stably expressed the *PvUPS1* promoter–*GUS* construct in soybean and analyzed the tissue-specific activity of the promoter in non-nodulated plants using histochemical GUS assays (Fig. 1). In leaves, GUS staining was detected in the minor as well as in the major veins (Fig. 1A–C). Higher magnification imaging of minor and major veins reported expression throughout the phloem as well as in the surrounding bundle sheath and vascular parenchyma cells (Fig. 1B, C). GUS expression was further found in the phloem and vascular parenchyma of stems and roots (Fig. 1D–G). Together, this expression pattern is in line with previous RNA localization studies and the predicted role of UPS1 in phloem loading and in N cycling between the xylem and phloem along the transport pathway (Pélissier and Tegeder, 2007; cf. Tegeder and Masclaux-Daubresse, 2018). The results also suggest that the *PvUPS1* promoter is suitable for manipulating phloem loading and source-to-sink partitioning of ureides in soybean using an *UPS1* overexpression approach.

In roots, *PvUPS1* expression was also reported in the pericycle, suggesting a function in N supply of lateral root sinks (Tegeder and Hammes, 2018). In addition, *PvUPS1* is expressed in the root endodermis (Fig. 1F, G; Pélissier and Tegeder, 2007). In non-nodulated soybean plants, *de novo* synthesis of allantoin and allantoic acid occurs in the root cortex, followed by their movement towards the vascular bundle. However, some of these ureides may leak into the cell wall space and need to be retrieved by UPS1 as the Casparian strip of the vascular endodermis blocks apoplastic movement to the root xylem (cf. Pélissier and Tegeder, 2007; Collier and Tegeder, 2012; Carter and Tegeder, 2016). *UPS1* expression in the endodermis is most probably enabling reuptake of apoplastic allantoin and allantoic acid into the symplasm to facilitate their movement to the xylem for long-distance transport to the shoot.

### PvUPS1 is a plasma membrane transporter and expressed in the root and shoot of the transgenic soybean plants

**Fig. 2.**
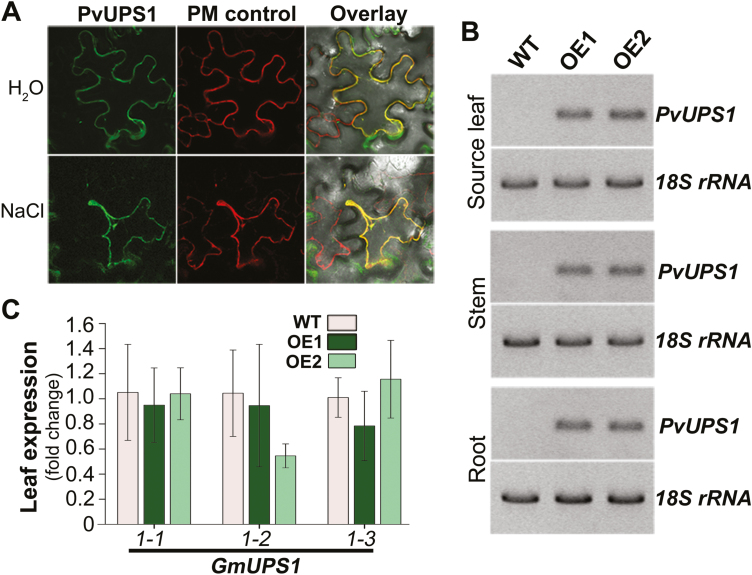
Membrane localization of common bean PvUPS1 and analysis of ureide transporter transcript levels in transgenic soybean plants expressing *PvUPS1* under control of the *PvUPS1* promoter (see Fig. 1). (A) Localization of GFP–PvUPS1 fusion proteins in leaf epidermal cells of *Nicotiana benthamiana*. The top row shows turgid cells. The bottom row shows NaCl-treated, plasmolyzed cells. The aquaporin (AtPIP2A)–mCherry fusion protein was used as control for plasma membrane localization. Plasma membrane co-localization of GFP–PvUPS1 and AtPIP2A–mCherry is shown in the overlay image. (B) Expression analysis of *PvUPS1* in source leaves, stems, and roots of soybean *UPS1* overexpressor lines OE1 and OE2 and wild-type plants (WT) using RT–PCR. (C) Expression of the three soybean ureide transporter genes *GmUPS1-1*, *1-2*, and *1-3* in source leaves of the OE lines using qPCR (quantitative real-time PCR). Data derive from at least four biological replicates (*n* ≥4) and were tested by Student’s *t*-test. Error bars depict ±SD.

Previous work supported a role for PvUPS1 in cellular import of ureides, but its membrane localization has not been resolved (Pélissier and Tegeder, 2007; Carter and Tegeder, 2016). Transporter–GFP fusion proteins were transiently expressed in *N. benthamiana* leaf cells and co-localized with a plasma membrane control (Fig. 2A; upper row). Microscopic analysis showed that PvUPS1 is targeted to the plasma membrane. The localization to the plasma membrane was even more evident when the tobacco cells were plasmolyzed (Fig. 2A; lower row).

To determine if *PvUPS1* is expressed in soybean plants transformed with the *PvUPS1* promoter–*PvUPS1* cDNA construct (Carter and Tegeder, 2016; *UPS1*-OE plant), its transcript levels were analyzed in source leaves, stems, and roots of two obtained *UPS1*-OE lines (Fig. 2B). *PvUPS1* expression was detected in all organs tested. Expression of the endogenous *GmUPS1* transporters was not changed compared with the wild-type control (Fig. 2C). Together with the *PvUPS1* promoter–GUS and membrane localization studies (Figs 1, 2A, B), the results suggest an overall increase in *UPS1* transporter expression in the *UPS1*-OE plants, most probably affecting phloem loading and source-to-sink partitioning of ureides.

### Phloem loading and root-to-leaf-to-sink partitioning of ureides are increased in *UPS1* overexpressors

**Fig. 3.**
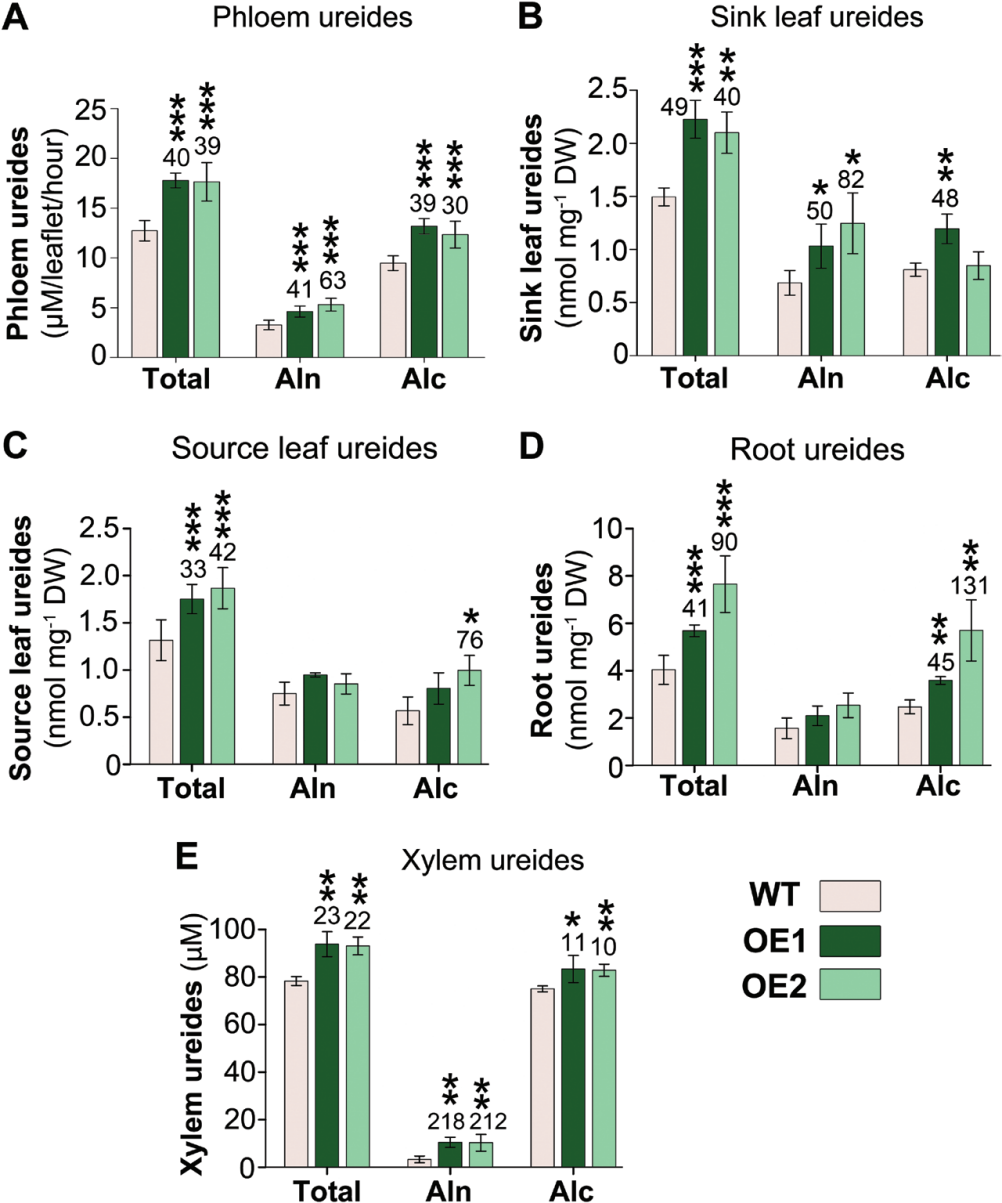
Analyses of total ureides, allantoin, and allantoic acid in (A) leaf phloem exudates (*n*=5), (B) sink leaves (*n* ≥3), (C) source leaves (*n* ≥3), (D) roots (*n*=3), and (E) xylem (*n* ≥3) of *UPS1-*overexpressing soybean lines (OE1 and OE2) and wild-type (WT) plants. Allantoin, Aln; allantoic acid, Alc. Data were tested by one-way ANOVA. Error bars depict ±SD, and asterisks indicate small, moderate, and large statistically significant changes from the WT (*P≤0.05, **P≤0.01, ***P≤0.001). Numbers above columns show the percentage change in *UPS1*-OE lines compared with the WT.

To determine if *UPS1* overexpression results in changes in phloem loading of ureides, leaf phloem exudates were analyzed. Depending on the overexpressor line, total ureide levels were elevated by up to 40%. This increase was due to a significant increase in both allantoin and allantoic acid (Fig. 3A). Ureide levels were also enhanced in *UPS1*-OE sink leaves (Fig. 3B), supporting increased source-to-sink allocation of the organic N. In addition, total ureide amounts were up-regulated in the transgenic source leaves (Fig. 3C), together suggesting improved delivery of root-synthesized ureides to the shoot. In fact, ureide levels in the roots (Fig. 3D) as well as in the xylem transpiration stream (Fig. 3E) of *UPS1*-OE plants were significantly increased.

### Root nitrogen metabolism and amino acid source-to-sink transport are increased

**Fig. 4.**
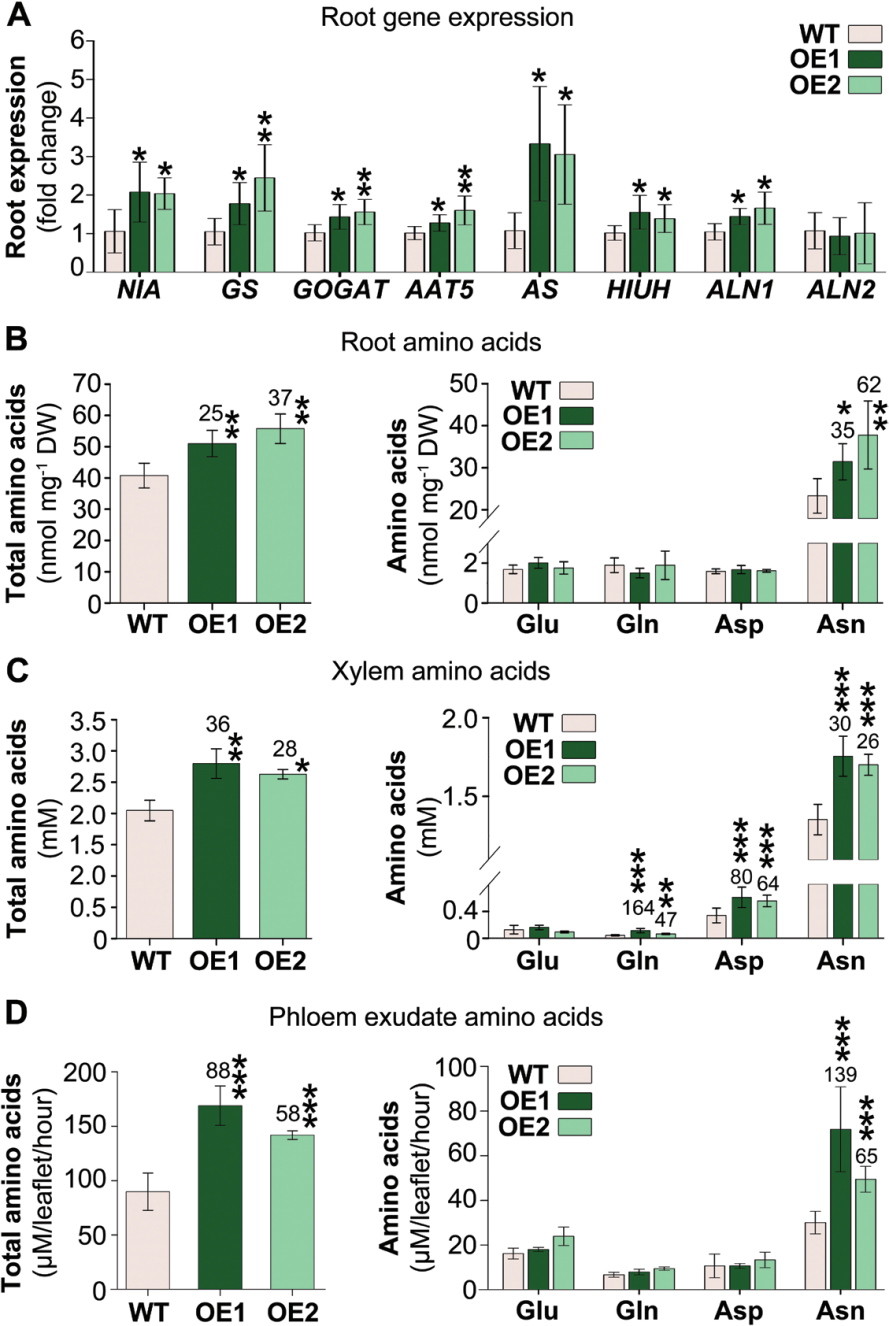
Effects of *UPS1* overexpression on root nitrogen metabolism and root-to-shoot-to-sink amino acid transport. Results are shown for *UPS1-*overexpressing soybean lines OE1 and OE2 and wild-type (WT) plants, and are presented as means ±SD, with SD indicated by error bars. Data were tested by Student’s *t*-test (A) or one-way ANOVA (B–D). Small, moderate, and large statistically significant changes from the WT are marked with asterisks (*P≤0.05, **P≤0.01, ***P≤0.001). Numbers above columns show the percentage change in *UPS1*-OE lines compared with the WT. (A) Expression of genes related to N assimilation, and amino acid and ureide synthesis was analyzed by qPCR in lines OE1 and OE2 and WT plants (*n* ≥6). The analyses included genes coding for nitrate reductase (*NIA*), and genes related to amino acid assimilation (*GS*, glutamine synthetase; *GOGAT*, 2-oxoglutarate amino transferase), synthesis of aspartate (*AAT*, aspartate aminotransferase) and asparagine (*AS,* asparagine synthetase), as well as synthesis of allantoin (*HIUH*, hydroxyisourate hydrolase) and allantoic acid (*ALN*, allantoinases). For gene accessions, primer sequences, and references, see Supplementary Table S1. Shown is the fold change in gene expression in *UPS1*-OE lines compared with the WT and relative to *SKIP16* expression. (B–D) Analysis of total free amino acids as well as the most abundant amino acids Glu, Gln, Asp, and Asn. (B) Root, (C) xylem sap, and (E) leaf phloem exudate amino acids. Results are from at least four biological repeats (*n* ≥4).

The observed higher root and xylem ureide levels in the *UPS*1-OE plants indicate that either overall more N is taken up, assimilated, and used for ureide synthesis, or more root N is channeled into ureide synthesis, potentially at the cost of amino acid production. Expression analysis of root genes related to N assimilation and ureide synthesis was performed (Fig. 4A). Results showed that transcript levels of genes encoding nitrate reductase (*NIA*) and amino acid assimilation (*GS*, glutamine synthetase; *GOGAT*, 2-oxoglutarate aminotransferase) were generally up-regulated in *UPS1*-OE roots. In addition, expression of genes involved in allantoin (*HIUH*, hydroxyisourate hydrolase) as well as allantoic acid synthesis (*ALN*, allantoinases), specifically *ALN1*, was increased. Further, transcript levels of genes essential for root synthesis of asparagine (*AAT*, aspartate aminotransferase; *AS*, asparagine synthetase), the main N transport compound in non-nodulated soybeans (Rainbird *et al.*, 1984), were significantly up-regulated in the transgenic plants. These data suggest that not only ureide synthesis but also amino acid synthesis is improved in *UPS1*-OE roots.

We examined if indeed altered ureide synthesis and allocation processes affect the steady-state pools of amino acids in *UPS*1-OE roots and if amino acid partitioning is also influenced by the changes (Fig. 4B–D). HPLC analysis showed a significant increase in root amino acids by 25–37% depending on the *UPS*1-OE line (Fig. 4B). This change was due to enhanced asparagine levels. Further, analysis of the xylem sap and leaf phloem exudates revealed a marked increase in amino acids in both transport pathways by up to 36% and 88%, respectively (Fig. 4C, D), supporting that root-to-leaf-to-sink transport of amino acids is up-regulated in the transgenic plants.

### Leaf nitrogen pools are changed

**Fig. 5.**
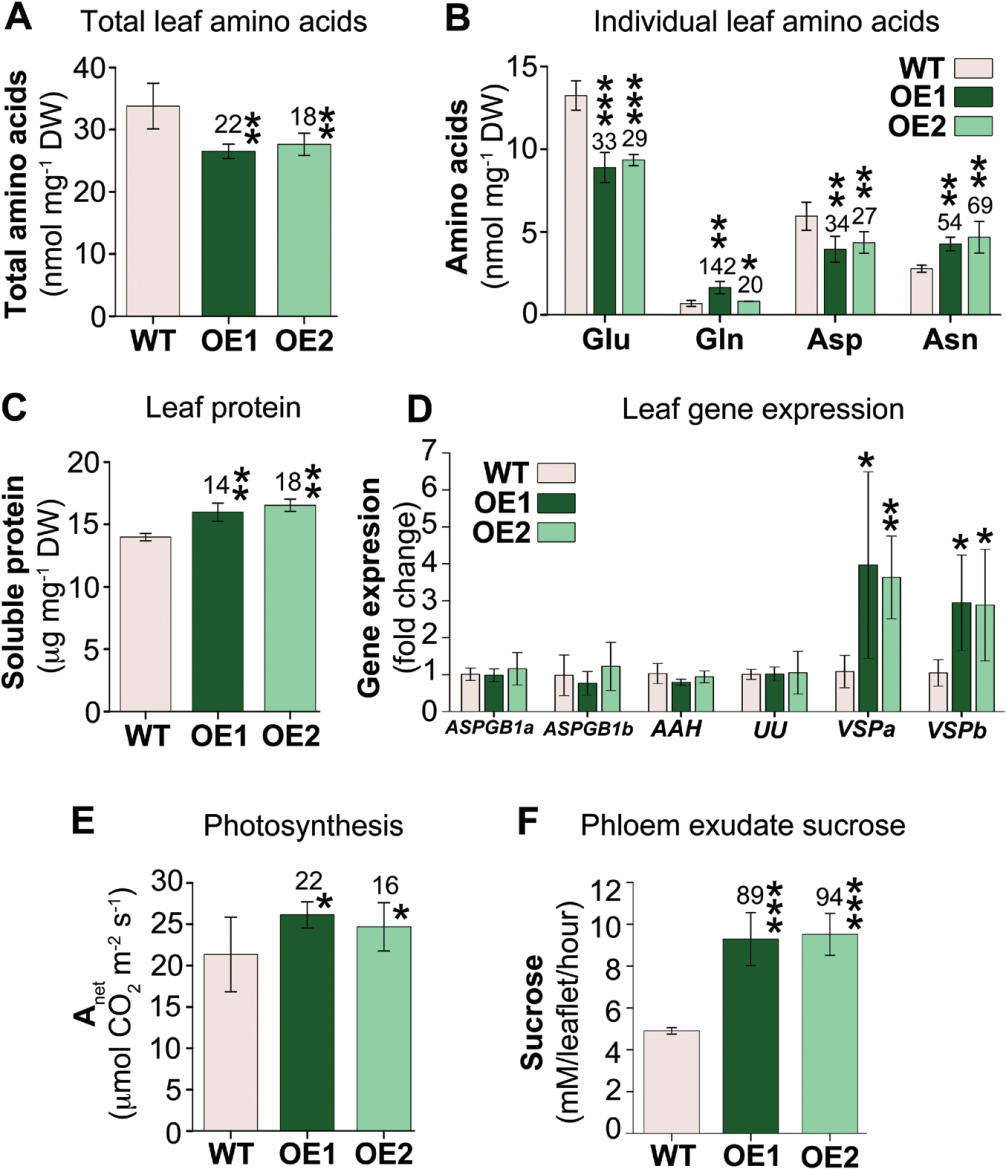
Effect of *UPS1* overexpression on leaf N status, C fixation, and source-to-sink sucrose partitioning. Results are shown for *UPS1-*overexpressing soybean lines OE1 and OE2 and wild-type (WT) plants, and are presented as means ±SD, with SD indicated by error bars. Data were tested by one-way ANOVA (A–C, E, F) or Student’s *t*-test (D). Small, moderate, and large statistically significant changes from the WT are marked with asterisks (*P≤0.05, **P≤0.01, ***P≤0.001). Numbers above columns show the percentage change in *UPS1*-OE lines compared with the WT. (A) Total free amino acid levels in source leaves (*n* ≥4). (B) Leaf levels of the most abundant amino acids Glu, Gln, Asp, and Asn (*n* ≥4). (C) Leaf soluble protein (*n* ≥4). (D) Expression analysis of leaf genes related to amino acid and ureide catabolism, and synthesis of vegetative storage proteins by qPCR. The analyses included genes coding for asparaginases (*ASPGB1a* and *ASPGB1b*), allantoate amidohydrolase (*AAH*), ubiquitous urease (*UU*), and vegetative storage proteins α and β (*VSPa* and *VSPb*) (*n* ≥4). For gene accessions, primer sequences, and references, see Supplementary Table S1. Shown is the fold change in gene expression in soybean lines overexpressing *UPS1* (OE) compared with the WT and relative to *SKIP16* expression. (E) Net CO_2_ assimilation rate (*n* ≥4). (F) Sucrose levels in leaf phloem exudates (*n*=5).

After arriving in source leaves, large amounts of ureides and amino acids are loaded into the phloem for N redistribution to developing sinks (see Figs 3A, 4D; Tegeder, 2014). However, organic N is also used for leaf metabolism, or it can be transiently stored during vegetative growth phase, as either ureides, amino acids, or VSPs to provide reproductive sinks with N during reproductive development (Franceschi *et al.*, 1983; Hsu *et al.*, 1984; Rainbird *et al.*, 1984; Costigan *et al.*, 1987; Staswick *et al.*, 2001). While ureides accumulated in *UPS1*-OE leaves (see Fig. 3C), total free amino acid levels were significantly down-regulated (Fig. 5A), primarily due to reductions in glutamate and aspartate that were the most abundant amino acids in leaves (Fig. 5B). Analysis of the total soluble protein showed a significant increase of up to 18% in the transgenic leaves (Fig. 5C), indicating that increased amounts of amino acids delivered to *UPS1*-OE source leaves (Fig. 4C) are used for improved protein synthesis rather than being transiently stored (cf. Fig. 5A). Asparagine is the dominant amino acid translocated in the xylem to soybean leaves (see Fig. 4C). Expression of *ASPGB* genes coding for asparaginases was not changed (Fig. 5D), implying that increased deamination of asparagine to mobilize N for enhanced protein synthesis in *UPS1*-OE leaves (Fig. 5C) may not be required. Similarly, expression levels of allantoate amidohydrolase (*AAH*) and ubiquitous urease (*UU*) genes involved in ureide catabolism were not changed, supporting that the additionally imported ureides are stored (see Fig. 3C, E) instead of being metabolized.

As mentioned above, in soybean leaves, N can also temporarily accumulate as VSP (Franceschi *et al.*, 1983; Franceschi and Giaquinta, 1983). Expression of genes encoding two abundant proteins VSPα and VSPβ was analyzed (Fig. 5D; cf. Staswick *et al.*, 2001). The results showed that both *VSPa* and *VSPb* are significantly up-regulated in *UPS1*-OE compared with wild-type leaves (Fig. 5D), suggesting that the observed higher levels of soluble protein in the transgenic leaves (see Fig. 5C) were, at least in part, due to increases in VSPs.

### Carbon fixation and source-to-sink transport are increased

Previous work in Arabidopsis and pea has shown that increased delivery of organic N to leaves can also result in increased pools of Rubisco (see Fig. 5C) and positively affect C assimilation (Zhang *et al.*, 2015; Perchlik and Tegeder, 2017, 2018). Indeed, analysis of photosynthesis showed a significant up-regulation of CO_2_ uptake in *UPS1*-OE leaves (Fig. 5E). Further, sucrose levels in phloem exudates of *UPS1*-OE leaves were enhanced by up to 94% (Fig. 5F), supporting that increased amounts of assimilated C are exported from leaves and allocated to sinks.

### Temporary nitrogen storage pools in stems are increased

**Fig. 6.**
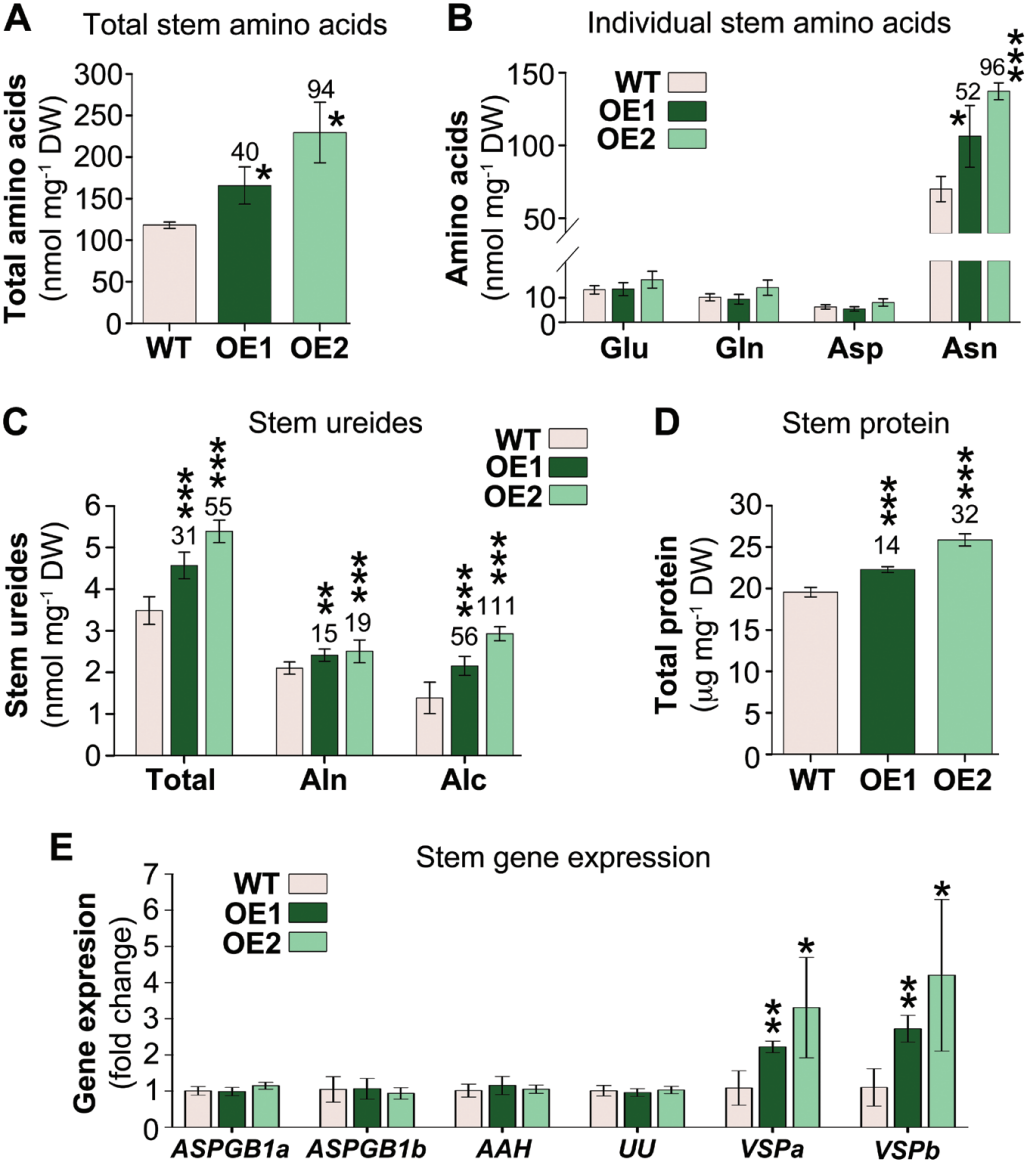
Effects of *UPS1* overexpression on N storage in the stem. Results are shown for *UPS1-*overexpressing soybean lines OE1 and OE2 and wild-type (WT) plants, and are presented as means ±SD, with SD indicated by error bars. Data were tested by one-way ANOVA (A–D) or Student’s *t*-test (E). Small, moderate, and large statistically significant changes from the WT are marked with asterisks (*P≤0.05, **P≤0.01, ***P≤0.001). Numbers above columns show the percentage change in *UPS1*-OE lines compared with the WT. (A) Total free amino acid levels in stem (*n* ≥3). (B) Stem levels of the most abundant amino acids Glu, Gln, Asp, and Asn (*n* ≥3). (C) Stem levels of total ureides, allantoin (Aln), and allantoic acid (Alc) (*n*=5). (D) Stem soluble protein (*n* ≥3). (E) Expression analysis of genes related to amino acid and ureide catabolism, and synthesis of vegetative storage protein in stem. qPCR was performed for genes encoding asparaginases (*ASPGB1a* and *ASPGB1b*), allantoate amidohydrolase (*AAH*), ubiquitous urease (*UU*), and vegetative storage proteins α and β (*VSPa* and *VSPb*) (*n* ≥4).For gene accessions, primer sequences, and references see Supplementary Table S1. Shown is the fold change in gene expression in *UPS1*-OE lines compared with the WT and relative to *SKIP16* expression.

Besides leaves, soybean stems also transiently store N during the vegetative phase to cover the sink N demand during reproductive development (Franceschi and Giaquinta, 1983; Franceschi *et al.*, 1983; Staswick, 1994). Analysis of organic N pools revealed a significant increase in amino acids (Fig. 6A), specifically asparagine (Fig. 6B), total ureides (Fig. 6C), and proteins (Fig. 6D), in *UPS1-OE* stems. When examining the expression of genes involved in asparagine deamination (*ASPGB1a* and *ASPGB1b*) and ureide degradation (*AAH* and *UU*), no changes were observed, indicating no differences in organic N catabolism in *UPS1*-OE versus wild-type stems (Fig. 6E). In contrast, transcript levels of VSP genes (*VSPa* and *VSPb*) were increased, suggesting that in the transgenic stems more amino acids are channeled into temporary protein storage pools. Together with the leaf analyses, the results also suggest that, compared with wild-type, overall more N is accumulated in *UPS1*-OE source leaves and stems during the vegetative stage (see Figs 3C, 5A–D, 6) to accommodate sink demands during reproductive growth.

### Total nitrogen levels are increased in stems and source leaves

**Fig. 7.**
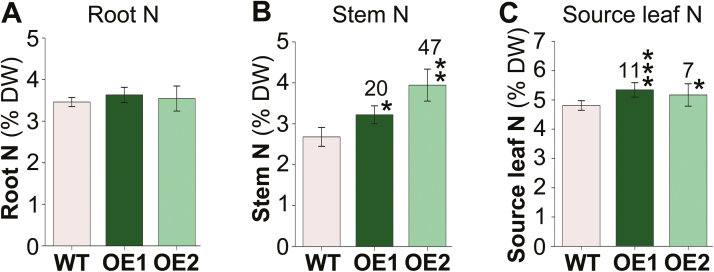
Analysis of elemental N in (A) roots, (B) stems, and (C) source leaves. Shown is the percentage (%) of N in dried tissues of *UPS1-*overexpressing soybean lines (OE1 and OE2) and wild-type (WT) plants (*n*=4). Data were tested by Student’s *t*-test. Error bars depict ±SD, and asterisks indicate small, moderate, and large statistically significant changes from the WT (*P≤0.05, **P≤0.01, ***P≤0.001). Numbers above columns show the percentage change in *UPS1*-OE lines compared with the WT.

Molecular and biochemical analyses of roots, stems, and leaves suggest that *UPS1*-OE plants take up, assimilate, and transiently store more N than the wild type (see Figs 3–6). To determine if the increased organic N response due to UPS1 is in fact linked to increased N uptake, elemental N levels in root, stem, and source leaf tissues were examined (Fig. 7). The results show no changes in %N in roots (Fig. 7A), while N levels in both stems and source leaves were significantly increased by up to 47% and 11%, respectively (Fig. 7B, C).

### Vegetative growth and seed development are improved

**Fig. 8.**
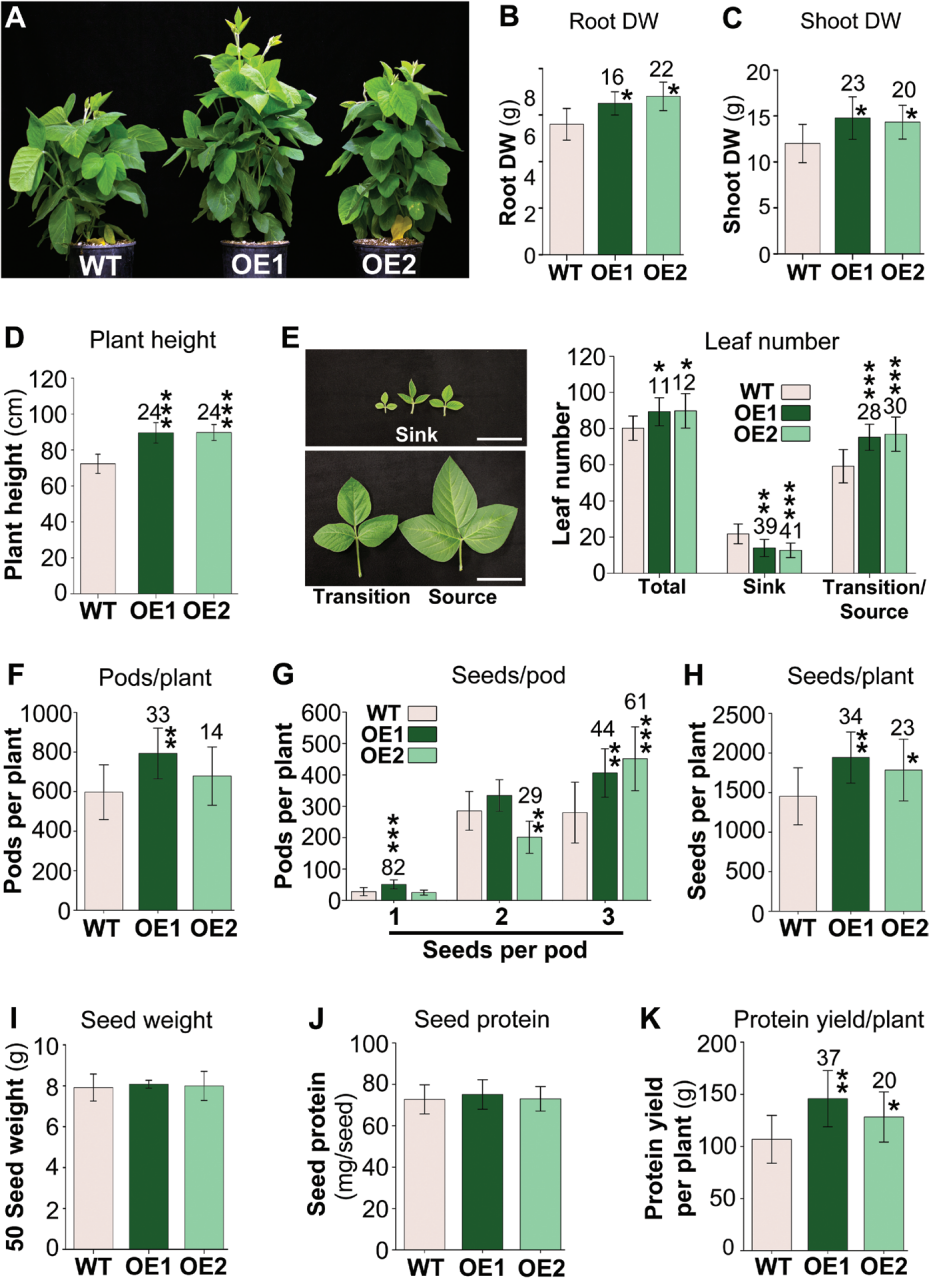
Effects of *UPS1* overexpression on plant growth and seed development. Results are shown for 36-day-old *UPS1-*overexpressing soybean lines OE1 and OE2 and wild-type (WT) plants, and are presented as means ±SD, with SD indicated by error bars. Data were tested by one-way ANOVA (B and C) or Student’s *t*-test (D–K). Small, moderate, and large statistically significant changes from the WT are marked with asterisks (*P≤0.05, **P≤0.01, ***P≤0.001). Numbers above columns show the percentage change in *UPS1*-OE lines compared with the WT. (A) Growth phenotype of *UPS1*-OE and WT plants. (B) Root and (C) shoot biomass (DW; *n* ≥8). (D) Plant height. (E) Analysis of total leaf number and total number of sink leaves as well as transition and source leaves (*n* ≥9). The image shows leaves that were classified as sink, sink-to-source transition, and source leaves. Sink leaves were defined as new, developing, and folded leaves. The unfolded leaves included expanding leaves that transition from sink to source (i.e. transition leaves) as well as fully expanded source leaves. Scale bars=5 cm. (F) Total number of pods per plant at desiccation/harvest (*n* ≥9). (G) Distribution of one-, two-, and three-seeded pods (*n* ≥9). (H) Total number of seeds per plant at desiccation (*n* ≥9). (I) Fifty-seed weight at desiccation (*n* ≥9 plants with five measurements per plant). (J) Amount of soluble protein per dry seed (*n* ≥9). (K) Total seed protein yield per plant (*n* ≥9).

Phenotypic analysis indicated that non-nodulated *UPS1*-OE plants grow bigger than the wild type (Fig. 8A). This was further examined by root and shoot biomass analysis. The results showed that the total *UPS1*-OE root dry weight was elevated by up to 22% (Fig. 8B). Since both *UPS1*-OE and wild-type soybean plants developed considerable root mass and at varying length, it was not possible to determine if this difference was due to more secondary roots, longer roots, or a greater root surface area (Supplementary Fig. S1). Analysis of shoot dry weight showed an increase between 20% and 23% dependent on the transgenic line (Fig. 8C). The *UPS1*-OE plants were 24% taller than wild-type plants (Fig. 8D) and developed significantly more leaves (Fig. 8E). We additionally determined the number of developing, yet folded sink leaves as well as the amount of unfolded leaves (Fig. 8E). The unfolded leaves included expanding leaves that transition from sink to source (i.e. transition leaves) as well as fully expanded source leaves. The results showed a decrease in sink leaf number in the transgenic plants, while numbers of transition and source leaves were significantly increased. These results support that leaf development is accelerated in the *UPS1*-OE plants, providing more leaf surface area for photosynthesis early in development.

We further analyzed if and how the changes in N and C metabolism and partitioning in *UPS1*-OE plants affect seed development. The results showed a tendency for an increase in pod number, but it was only significant for the *UPS1*-OE1 line (Fig. 8F). However, *UPS1*-OE1 and *UPS1*-OE2 plants developed significantly more pods that carried three seeds compared with one- or two-seeded pods (Fig. 8G). This resulted in an overall increase in the total seed number per transgenic plant of up to 34% (Fig. 8H). Seed weight was not changed in the *UPS1*-OE lines (Fig. 8I), which is consistent with no changes in soluble protein per seed (Fig. 8J). When calculating the total seed protein yield per plant, also referred to as harvestable protein, the results showed increases in seed protein yields between 20% and 37% dependent on the transgenic line (Fig. 8K). Together, the results show that the combined changes in vegetative biomass, assimilation, and photoassimilate partitioning in *UPS1*-OE plants compared with the wild type lead to improved seed development and thereby higher seed protein yield.

## Discussion

### UPS1 function in the leaf phloem is essential for ureide partitioning to sinks

Transport of ureides and other assimilates to developing sinks generally occurs from leaves to sinks in the phloem (Atkins and Smith, 2007; Tegeder, 2014). In this study, expression of common bean *PvUPS1* in the leaf phloem of non-nodulated soybean plants resulted in enhanced leaf-to-sink partitioning of allantoin and allantoic acid, as indicated by their increased levels in *UPS1*-OE phloem exudates (Fig. 3A) and sink leaves (Fig. 3B). Previous studies with yeast (*Saccharomyces cerevisiae*) expressing *PvUPS1* suggested that PvUPS1 transports allantoin but not allantoic acid (Pélissier *et al.*, 2004), in contrast to soybean GmUPS1-1 and GmUPS1-2 that mediate transport of allantoin and allantoic acid (Collier and Tegeder, 2012). However, since leaf expression of the endogenous *GmUPS1* transporter genes is not changed in *UPS1-*OE compared with wild-type plants (Fig. 2C), it is reasonable to assume that the increased translocation of the two ureides is due to PvUPS1 function in both allantoin and allantoic acid transport, and that the yeast studies do not fully reflect the physiological function of PvUPS1 (Pélissier *et al.*, 2004; cf. Carter and Tegeder, 2016). The concurrent leaf expression of *PvUPS1* and *GmUPS1* transporter genes (Fig. 2B, C) further suggests that in soybean the *PvUPS1* promoter functions in a similar manner to the endogenous *UPS1* promoters; however, this remains to be examined in future experiments.

In *UPS1*-OE plants, xylem concentrations of allantoin and allantoic acid were also improved (Fig. 3E), suggesting that the increased amounts of phloem and sink ureides were derived from *de novo* allantoin and allantoic acid synthesis in roots and their subsequent root-to-leaf translocation. This corresponds to the up-regulated expression of genes involved in root allantoin and allantoic acid synthesis (Fig. 4A) and with increased ureide levels in *UPS1*-OE roots (Fig. 3D). Overall, the results support that UPS1 transporter function in the leaf phloem presents a bottleneck in source-to-sink allocation of ureides and influences allantoin and allantoic acid levels in source and sink (Fig. 3). The data also suggest that even though tissue, xylem, and phloem levels of allantoin and allantoic acid in non-fixing soybean plants are relatively low compared with amino acids (Figs 3–6; cf. Matsumoto *et al.*, 1977; McClure and Israel, 1979; McNeil and LaRue, 1984; Do Amarante *et al.*, 2006), ureides still contribute to source-to-sink partitioning of N and sink N nutrition. Similarly, recent studies with non-nodulated, N-fed common bean plants imply that ureides from recycling of leaf N add to sink N supply (Díaz-Leal *et al.*, 2012). This further agrees with work in non-legume plants including Arabidopsis and rice, which also possess ureide biosynthetic and catabolic genes and can utilize ureides when present as a sole N source (Desimone *et al.*, 2002; Yang and Han, 2004; Brychkova *et al.*, 2008; Werner *et al.*, 2008; Lee *et al.*, 2018). Indeed, under low N conditions, Arabidopsis up-regulates *UPS* transporter gene expression to facilitate remobilization and transport of ureides from leaves to sinks (Soltabayeva *et al.*, 2018). Moreover, UPS transporter function generally seems to be required for development as *ups* mutants display reduced vegetative growth and early transition to reproductive phase (Takagi *et al.*, 2018).

### Ureide phloem loading affects root N assimilation and root-to-shoot translocation of amino acids

Enhanced ureide production in *UPS1*-OE roots could potentially compete with N utilization for synthesis of amino acids and especially asparagine, which is the principal N assimilate found in the xylem and phloem transport pathways in non-nodulated soybean (Fig. 4C, D; Rainbird *et al.*, 1984; Lea *et al.*, 2007). However, in *UPS1*-OE roots, expression of amino acid synthesis genes was up-regulated (Fig. 4A) and amino acid levels, particularly those of asparagine, were increased in roots, xylem, and phloem (Fig. 4B–D). This suggests that changes in ureide phloem loading not only positively influence ureide production and source-to-sink partitioning, but also enhance N assimilation for amino acid synthesis and distribution to sinks. Although the molecular basis remains to be elucidated, these modifications in the N household and N partitioning processes in *UPS1*-OE plants may be triggered through changes in cellular or organ levels of ureides (or related N compounds) and be regulated via feedback control (Tegeder and Masclaux-Daubresse, 2018). A similar regulatory effect has been observed in pea plants where increased phloem loading of *S*-methylmethionine (SMM) led to an up-regulation of SMM and amino acid synthesis, as well as increased source-to-sink translocation of organic sulfur and N (Tan *et al.*, 2010). In addition, overexpression of a broad specific amino acid transporter in the pea phloem resulted in enhanced N partitioning to sinks, which in turn positively affected N uptake from the soil, amino acid synthesis in roots, and root-to-shoot N allocation (Zhang *et al.*, 2015). A regulatory or signaling function of ureides is also in line with recent studies in Arabidopsis indicating that allantoin influences plant physiological processes related to environmental stress by affecting distinct biochemical pathways (Brychkova *et al.*, 2008; Irani and Todd, 2016; Takagi *et al.*, 2016). For example, elevated allantoin levels result in activation of hormone metabolism and thereby control plant responses to drought (Watanabe *et al.*, 2014) or biotic stress (Takagi *et al.*, 2016).

### UPS1 function contributes to ureide distribution and temporary storage along the transport pathway from root to shoot

Previous work has demonstrated that organic N may exit the xylem stream and be transferred to the transport phloem for immediate sink N supply (Pate *et al.*, 1975; van Bel, 1984, 1990; Atkins and Smith, 2007). Studies on amino acid transport have shown that this xylem-to-phloem exchange involves regulated lateral movement via parenchyma cells and a phloem loading step, both requiring plasma membrane-bound importers, namely in the vascular parenchyma cells as well as the SE–CC (sieve element–companion cell) complex (Hunt *et al.*, 2010; Zhang *et al.*, 2010). Based on *UPS1* expression in these cell types in stem and leaf major veins (Fig. 1; Pélissier and Tegeder, 2007), it is therefore fair to speculate that in the *UPS1*-OE plants enhanced xylem-to-phloem transfer of ureides also contributes to the increased shoot sink N supply. As indicated by *UPS1*–GUS expression in the root phloem and vascular parenchyma (Fig. 1F, G), this exchange between the xylem and phloem may also occur in roots to facilitate prompt ureide delivery for lateral root development and tip growth (Fig. 8B; Supplementary Fig. S1).

During vegetative growth of soybean, large amounts of ureides can be found in stems as well as in leaves, especially in vacuoles of bundle sheath and paraveinal mesophyll cells (Pate, 1980; Layzell and LaRue, 1982; Franceschi *et al.*, 1983; Rainbird *et al.*, 1984; Costigan *et al.*, 1987; Kaschuk *et al.*, 2010). In addition, relatively high quantities of N may also accumulate in source leaves and/or stems in short-term storage pools of amino acids, specifically asparagine, or VSPs (Hanway and Weber, 1971; Atkins *et al.*, 1975; Franceschi *et al.*, 1983; Wittenbach *et al.*, 1984; Everard *et al.*, 1990; Lea *et al.*, 2007). Indeed, in leaves and stems of *UPS1-*OE plants, ureide (Figs 3C, 6C), asparagine (Figs 5B, 6B), and protein/*VSP* levels (Figs 5C, D, 6D, E) were strongly elevated. This suggests that during the vegetative phase of *UPS1*-OE compared with wild-type plants, N assimilation greatly exceeds the N demand for growth, which results in modifications in N fluxes and increased temporary storage pools of diverse forms of N (Millard, 1988; Staswick, 1994; Volenec *et al.*, 1996). What is regulating the size of the different storage pools is unknown, but an important function of VSPs seems to be sequestering an excess of amino acids, potentially to avoid toxic or inhibitory effects, as well as in N remobilization when sink N requirements are high, as is the case during the reproductive phase (Franceschi *et al.*, 1983; Staswick, 1994). Clearly, improved ureide partitioning in *UPS1*-OE plants leads to an increase in temporary N storage, thereby most probably providing more N for remobilization during pod and seed development (Fig. 8F–H).

### Increased assimilate partitioning from source to sink affects soybean growth and sink development

The overall outcome of increased phloem loading with ureides was an improved vegetative growth as well as elevated seed development (Fig. 8A–H). The contribution of allantoin and allantoic acid to this improvement seems to be 2-fold. On one hand, their increased translocation in the *UPS1*-OE phloem added to enhanced sink N nutrition and growth. Even though the amount of phloem amino acids was much higher than that of ureides (Figs 3A, 4D), an increase in ureides strongly affects the N content due to their N to C ratio of 1:1 compared with asparagine of 1:2. Furthermore, as discussed above, numerous studies support that allantoin plays diverse roles in plant physiology, especially in regulating responses to environmental stresses including N limitation (Soltabayeva *et al.*, 2018; Redillas *et al.*, 2019). Our studies suggest that changes in ureide or allantoin pools in the SE–CC complex, or its close vicinity (e.g. apoplast or parenchyma/bundle sheath cells), result in modifications in N and C acquisition and metabolism, and in the rebalancing of whole-plant ureide, amino acid, and sucrose partitioning (Figs 3–6). Together, these complex alterations in the N and C status throughout the *UPS1-*OE plants and ultimately the increased metabolite availability in the phloem most probably trigger improved seed development in soybean (cf. Herman, 2014; Santiago and Tegeder, 2016, 2017). Similar, multifaceted adjustments in the C and N (and/or sulfur) household and source-to-sink transport have been observed in pea when phloem loading of amino acids or sucrose was up-regulated (Zhang *et al.*, 2015; Lu *et al.*, 2020), potentially hinting at the presence of a common scheme of signaling, sensing, and modifying the C/N balance upstream of C and N phloem loading.

The production of high-yielding soybean plants has been a difficult challenge as there is generally a strong negative correlation between seed yield and protein concentration (Openshaw *et al.*, 1979; Schonbeck *et al.*, 1986, Wilcox and Shibles, 2001; Proulx and Naeve, 2009). However, this negative relationship was not observed in *UPS1*-OE plants that showed an increase in seed number but no change in protein levels (Fig. 8G, H, J). This suggests that the N and C demand for an increased number of seeds as well as per seed could be accommodated through the increased source-to-sink partitioning of ureides, amino acids, and sucrose. It further indicates that the *UPS1*-OE plants use N more efficiently for seed yield and seed protein yield production than the wild type. The increase in N use efficiency in *UPS1*-OE plants is most likely to be due to an increase in N uptake efficiency as root N assimilation and total N amounts in shoot tissues were significantly increased (Figs 3D, 4A, B, 7). In addition, an increase in N utilization efficiency may further contribute to the higher seed yields in *UPS1*-OE plants, and future studies will need to address if the increased shoot ureide, amino acid, and protein storage pools established during vegetative growth (Figs 3C, 5A–D, 6) are effectively remobilized and efficiently utilized for reproductive development. Positive effects on N use efficiency have also been observed in transgenic pea lines, in which increased source-to-sink transport of amino acids led to improved seed yield with no change or an increase in seed protein levels, depending on whether moderate or high amounts of N were fertilized (Tan *et al.*, 2010; Zhang *et al.*, 2015; Perchlik and Tegeder, 2017). In two of those studies, not only was amino acid phloem loading engineered but the import of amino acids into individual pea seeds was also enhanced with the goal of simultaneously addressing source and sink limitations of seed yield productivity (Zhang *et al.*, 2015; Perchlik and Tegeder, 2017; cf. Herman, 2014; Rossi *et al.*, 2015; Burnett *et al.*, 2016). The concurrent up-regulation of N import into the phloem and embryo may also be an interesting strategy for soybean to improve seed number as well as seed protein amounts. As ureides are catabolized in the seed coat to ammonium for re-assimilation of amino acids (Winkler *et al.*, 1988; Todd *et al.*, 2006), which are subsequently taken up by the embryo (Hsu *et al.*, 1984; Rainbird *et al.*, 1984; Tegeder, 2014), such an approach would require overexpression of *UPS1* in the soybean phloem in tandem with overexpression of an amino acid transporter in the embryo.

### Conclusions

Non-fixing soybean plants use amino acids as main N forms but ureides also contribute to source-to-sink N allocation. Our data provide evidence that UPS1 transporter function in the phloem presents a bottleneck in long-distance transport and sink supply of ureides. They further support that UPS1-mediated phloem loading triggers, probably by a feedback regulatory signaling pathway, N uptake and ureide synthesis in roots, as well as ureide movement, compartmentation, and storage in the shoot of non-fixing, and most probably also N-fixing plants. In addition, the results hint at a complex inter-relationship between ureide transport and N/C households, and suggest that ureides occupy a crucial position in controlling amino acid and sucrose metabolism and partitioning, and C–N interactions in plants. The homeostasis of ureides, especially allantoin, or a co-regulated compound, might serve as a central switch that, when altered, will cause whole-plant level adjustments in N and C acquisition, assimilation, and transport for optimum plant growth. Such complex rebalancing may lead, at least in *UPS1*-OE plants, to enhanced growth and improved seed development.

Water is the main limiting environmental factor contributing to reduced productivity in N-fixing and non-fixing soybean (Serraj *et al.*, 1999; King and Purcell, 2005; Kirova *et al.*, 2008; Kunert *et al.*, 2016). Since ureides are predicted to have a regulatory role in plant drought stress tolerance (Silvente *et al.*, 2012; Yobi *et al.*, 2013; Watanabe *et al.*, 2014; Irani and Todd, 2016), it will be interesting to analyze the *UPS1*-OE plants under water deficit conditions and resolve whether the changes in ureide partitioning processes and homeostasis result in their adaptation to drought stress.

## Supplementary data

Supplementary data are available at *JXB* online.

Table S1. Primers used for gene expression analyses.

Fig. S1. Representative images of roots from three individual plants of the wild type (WT) and *UPS1*-overexpressing lines OE1 and OE2.

eraa146_suppl_Supplementary_Material
