# Supplementary material for: Role of ureides in source-to-sink transport of photoassimilates in non-fixing soybean
Source: J Exp Bot. 2020 Mar 19;71(15):4495–511. doi: 10.1093/jxb/eraa146 (PMC7475099; doi:10.1093/jxb/eraa146)

Table S1. Primers used for expression analyses.

Soybean and common bean accessions	Gene name	Function	Forward primer '5-3'<br>Reverse primer '5-3'	References
Glyma01g07120	*GmUPS1-1*	Ureide permease 1-1	GGAGACATCTCTTCCAAGGAGGGG<br>AGCAAAGTCCAGCAAAGAATGTTAGAGC	Collier and Tegeder (2012) The Plant Journal (2012) 72: 355–367.
Glyma02g12970	*GmUPS1-2*	Ureide permease 1-2	GGTGATTATAAAAATGGAGACACCTCTTTG<br>GAAGCAAAGTCCAGCAAAGAAAGTTAGAAA	Collier and Tegeder (2012) The Plant Journal (2012) 72: 355–367.
Glyma02g12980	*GmUPS1-3*	Ureide permease 1-3	CCTGTCTTAAACTTACCCAAGTCATCACTG<br>ACAAAACAATGCCCCAAAAGGTACTC	Collier and Tegeder (2012) The Plant Journal (2012) 72: 355–367.
AY461734	*PvUPS1*	Ureide permease	GTAAGCACTTTCTGGGGTGTTGTTCTA<br>CTTGGCAGTGTGAGAACTGCTTCTG	Pélissier et al. (2004) Plant Physiology 134: 664–675.
Glyma06g11430	*NIA*	Nitrate reductase	TTCATCAGAGGCTATGCGTATTCCG<br>CTTTTCAGGTTGGGTGTTGAGGG	Wu et al. (1995) Plant Molecular Biology 29: 491-506.
Glyma11g33560	*GS1*	Glutamine synthetase 1	CTATTCCCACTAACAAGAGGCACGC<br>GCCAACACCACAGTAGTATGGACC	Morey (2002) Plant Physiology 28: 182-193.
Glyma03g28410	*GOGAT*	Glutamate synthase	ACACTCTCATCTTCTCTCTCTCTCTCTCTCG<br>CGTTGTGGAGGGAAGGGAATG	O'Rourke (2014) Annals of Botany 113: 1107-1120.
Glyma17g33050	*AAT5*	Aspartate amino-transferase 5	GCTCAATCTTACAGTAAAAATCTCGGTCTC<br>TTCCAACAACATCGGCAACTATCC	Wadsworth (1993) Plant Molecular Biology 21: 993–1009.
Glyma18g06840	*AS*	Asparagine synthase	CTGTATGAGGAGCACGGAGAAAACTTTA<br>TCATCATTCAACCCCTTCAATTCAGAG	Hughes et al. (1997) Plant Molecular Biology 33:301-311.
Glyma14g09510	*ASPGB1a*	Asparaginase	GCGCGGCAGCAACATCCACG<br>CTTCACCCGTGCACGATACGCC	Pandurangan et al. (2012) Journal of Experimental Botany 63: 3173-3184.
Glyma17g35650	*ASPGB1b*	Asparaginase	CGGCAGCGACATCCACAGGG<br>CTCGCCCGTGCACGATACACC	Pandurangan et al. (2012) Journal of Experimental Botany 63: 3173-3184.
Glyma02g02230	*HIUH*	Hydroxyisourate hydrolase	TTGGAGAGGATCTGTTTTCAAATGAAGAG<br>GTGACGAATTACTTGCTGTCCGTTGAC	Raychaudhuri and Tipton (2002) 130:2061-2068.
Glyma15g07910	*ALN1*	Allantoinase 1	TATCCTACAACCGTGTCAAAGGAAAAACTA<br>AAGAACACCAGCACTTAAGAGCCCC	Duran and Todd (2012) Plant Physiology and Biochemistry 54: 149-155.
Glyma13g31430	*ALN2*	Allantoinase 2	TTACGAAGGACACAAGAAAAGGTGGC<br>GACAAGTCTCAACACTTATGCTGTCACCAT	Duran and Todd (2012) Plant Physiology and Biochemistry 54: 149-155.
Glyma15g16870	*AAH*	allantoate amidohydrolase	GATATACGAGCGATAGATGACCTTGGACG<br>CTTAGATCCGAATCACAAATCACAGCAC	Werner et al. (2008) Plant Physiology 146: 418-430.
Glyma11g37250	*UU*	ubiquitous urease	AGCATTTGGCAAGGCTGGTAGTTC<br>ATTCAGTTTCATGTCTCGTTTGGTGAGC	Goldraij et al. (2003) Plant Physiology 132:1801–1810.
Glyma07g01730	*VSPa*	Vegetative storage protein α	TTAAAGAAGGCTGGCTACCACACATG<br>GGTCCTGCTTTCGCCTCTGTGG	Mason et al. (1988) Plant Molecular Biology 11: 845–856.
Glyma08g21410	*VSPb*	Vegetative storage protein β	GGCATAGATAACACCGTACTCTCTAATATCCC<br>CCCATTCATCATATAAGGTTTCATTAAATTCC	Mason et al. (1988) Plant Molecular Biology 11: 845–856.
Glyma18g52780	*ACT11*	Cytoskeletal Actin	CGGTGGTTCTATCTTGGCATC<br>GTCTTTCGCTTCAATAACCCTA	Hu et al. (2009) BMC Molecular Biology 10: 93–104.
Glyma12g05510	*SKIP16*	SKP1/ASK-interacting protein 16	CTAATGGCAATTGCAGCTCTC<br>AGATAGGGAAATTGTGCAGGT	Hu et al. (2009) BMC Molecular Biology 10: 93–104.

Figure S1. Representative images of roots from (A to C) three individual plants of wild-type (WT) and *UPS1*-overexpression lines OE1 and OE1. Bars = 5 cm.

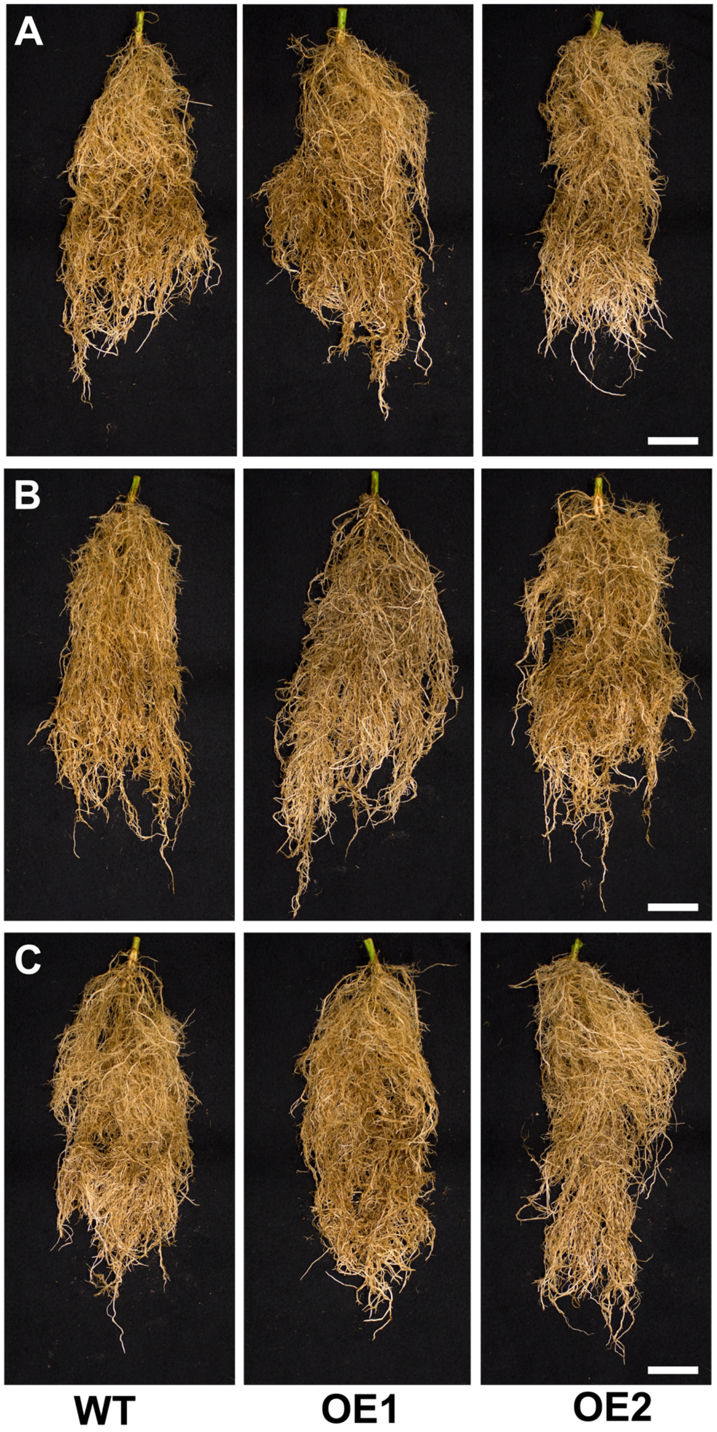

Supplement: eraa146_suppl_Supplementary_Material [file eraa146_suppl_supplementary_material.pdf]
